# Central and peripheral effects of physical exercise without weight reduction in obese and lean mice

**DOI:** 10.1042/BSR20171033

**Published:** 2018-03-05

**Authors:** Francine Pereira de Carvalho, Thaís Ludmilla Moretto, Izabelle Dias Benfato, Marcela Barthichoto, Sandra Mara Ferreira, José Maria Costa-Júnior, Camila Aparecida Machado de Oliveira

**Affiliations:** 1 Programa de Pós-Graduação Interdisciplinar em Ciências da Saúde, Universidade Federal de São Paulo, Santos, São Paulo, Brazil; 2 Programa de Pós-Graduação em Alimentos, Nutrição e Saúde, Universidade Federal de São Paulo, Santos, São Paulo, Brazil; 3 Departamento de Biologia Funcional e Estrutural, Instituto de Biologia, Universidade Estadual de Campinas (Unicamp), Campinas, São Paulo, Brazil; 4 Departamento de Biociências, Instituto de Saúde e Sociedade, Universidade Federal de São Paulo, Santos, São Paulo, Brazil

**Keywords:** energy balance, hypothalamus, insulin, leptin, obesity, physical exercise

## Abstract

To investigate the central (hypothalamic) and peripheral effects of exercise without body weight change in diet-induced obesity (DIO). Twelve-week-old male C57Bl/6 mice received a control (C) or a high-fat diet (H). Half of them had free access to running wheels for 5 days/week for 10 weeks (CE) and HE, respectively). Hypothalamic expression of genes related to energy homeostasis, and leptin (Stat3 and p-Stat3) and insulin (Akt and p-Akt) signaling were evaluated. Glucose and leptin tolerance, peripheral insulin sensitivity, and plasma insulin, leptin and adiponectin were determined. Perigonadal and retroperitoneal fat depots were increased by diet but reduced by exercise despite lack of effect of exercise on body weight. Blood glucose during intraperitoneal glucose tolerance test (ipGTT) was higher and glucose decay during intraperitoneal insulin tolerance test (ipITT) was lower in H and HE compared with C and CE. Exercise increased liver p-Akt expression and reduced fast glycemia. High-fat diet increased plasma insulin and leptin. Exercise had no effect on insulin but decreased leptin and increased adiponectin. Leptin inhibited food intake in all groups. Hypothalamic total and p-Stat3 and Akt were similar amongst the groups despite higher plasma levels of leptin and insulin in H and HE mice. High-fat diet modulated gene expression favoring a positive energy balance. Exercise only marginally changed the gene expression. Exercise induced positive changes (decreased fast glycemia and fat depots; increased liver insulin signaling and adiponectin concentration) without weight loss. Thus, despite reducing body weight could bring additional benefits, the effects of exercise must not be overlooked when weight reduction is not achieved.

## Introduction

Between 1980 and 2013, the percentage of overweight and obese has increased 27.5% in adults and 47.1% in children and adolescents, going from 857 million in 1980 to 2.1 billion in 2013 [1]. These data make clear the importance of creating strategies for combating this epidemic. Together with diet, exercise is one of the most used non-pharmacological strategies in the fight against obesity, but the results can be disappointing [2–6]. Exercise can elicit some behavioral and non-behavioral compensatory mechanisms making it difficult to achieve the expected body weight reduction [3,6,7].

According to the theory of ‘constrained-energy expenditure’, physical activity does not increase total energy expenditure (EE) in a dose-dependent manner. Instead, EE increases with physical activity at low levels but plateaus at higher levels as an evolved mechanism to maintain EE within a physiological narrow range [7]. In-line with this theory, we and others have shown that exercise can decrease non-exercise activities [2,6,8]. This reduction can be such that the total daily EE can remain unchanged despite the exercise done [2,9]. Also, exercise might increase energy intake [6,10,11]. Together, these compensatory mechanisms can explain the usually below-than-expected long-term effects of exercise for body weight control.

On the other hand, sedentary behavior increases the risk for metabolic and cardiovascular diseases even in those meeting the WHO recommendations for physical activity [12]. Thus, despite the reduction in body weight is a desirable outcome, a shift in focus from weight loss to improvements in exercise/physical activity and also diet has been suggested to manage obesity [13]. This ‘fat but fit’ idea, however, is far from being a consensus. Whereas some authors have found that the hazardous health effects of adiposity may be counterbalanced by an improvement in cardiorespiratory fitness (CRF) [14,15], Nordström et al. (2015) have recently shown that unfit normal-weight individuals had lower risk of death from any cause than did fit obese individuals [16].

Running wheels are widely used to analyze the effects of exercise on different conditions, including diet-induced obesity (DIO) [4,17]. However, weight loss is usually not observed with the use of activity wheels, as found by us and others [4,6,10]. Taking advantage of this, our purpose was to determine, in DIO mice, the isolated effect of exercise (without body weight modification) on insulin and leptin action on hypothalamus, on peripheral insulin sensitivity, and glucose homeostasis, all of which are negatively affected by obesity.

## Methods

### Animals

The experiments were approved by the Institutional Ethics Committee on Animal Use (CEUA 5042110514). Eight-week old male C57Bl/6 mice were obtained from the Center for Development of Animal Models for Medicine and Biology (CEDEME, Federal University of São Paulo). They were kept in collective cages (3–4 mice each) at the animal house of the Department of Bioscience in a temperature-controlled room (22°C) with a 12:12-h light/dark cycle (7:00–19:00 h) and had free access to a control diet with a caloric composition of 16.5% fat, 65.7% carbohydrate, and 17.7% protein and, a caloric density of 3.82 kcal/g. After a 4-week adaptation period, 12-week-old mice were randomly assigned into one of two diet groups for 10 weeks: they were either kept on the same control diet (C group) or fed a high-fat diet (H group) with a caloric composition of 60.2% fat, 26% carbohydrate, and 13.8% protein, and a caloric density of 5.23 kcal/g. In both the diets, the source of carbohydrate was maize starch, dextrinized maize starch, and sucrose; protein was from casein; and fat was from soybean oil. In the high-fat diet, there was the addition of lard. Half of the mice in both C and H groups stayed in individual cages with free access to a running wheel for 5 days a week (CE and HE groups, respectively). In the resting days (2 days/week), they returned to collective cages always with the same cage mates. To alleviate any stress caused by individual housing of CE and HE mice in the exercise days, even with the access to the running wheel, they were housed close to each other [18]. All diets were purchased from Rhoster (Rhoster Indústria e Comércio LTDA, SP, Brazil). The study was performed in three independent sets of experiments, and analysis were performed as follows: hypothalami for real-time PRC array were from mice of set 1; hypothalamic and liver insulin signaling experiments were performed in mice from set 2; hypothalamic leptin signaling and leptin tolerance test were done in mice from set 3. Plasma hormone concentration was determined in mice of sets 1 and 3. Each set had *n*=4–5 mice/group (total *n*=12–15 mice/group).

### Body weight, energy intake, and fat depots

Body weight was recorded once a week during all the experiments using a semi-analytical balance (Shimadzu©). Energy intake was measured in weeks 5 and 10, and during this measurement all mice were housed individually. Average daily consumption was determined by subtracting the weight of the remaining food removed after 48 h from the weight of food given, with care taken to account for spillage. The depots of retroperitoneal and perigonadal adipose tissue of all the mice were collected and weighed at the end of the experiment as an additional measure to assess obesity.

### Voluntary exercise

Mice were housed individually in home cages equipped with a running wheel (Panlab-Harvard Apparatus, Barcelona, Spain) for 10 weeks, 5 days a week, and rested for 2 consecutive days every week. The wheel (diameter: 34.5 cm; width: 9 cm) was mounted outside the home cage to preserve animal life space. The total number of wheel rotations was registered daily on an external LE907 individual counter.

### Intraperitoneal insulin and glucose tolerance tests

Both tests were performed 48 h after the last access to the running wheel for the CE and HE groups to avoid any acute effect of exercise. The intraperitoneal insulin tolerance test (ipITT) was performed in mice fasted for 6 h. Food was withdrawn at 7:00. Mice were injected i.p. with 0.5 U/kg body weight of human insulin (Biohulin N, Biobrás, Brazil). Blood samples were collected from the tip of the tail immediately before and 4, 8, 12, and 16 min after insulin injection for glucose analysis. For intraperitoneal glucose tolerance test (ipGTT), food was withdrawn at 7:00 and a fast blood sample was taken after 8 h. Subsequently, each mouse received an i.p. glucose solution load (2 g/kg body weight), and additional blood samples were collected at 15, 30, 60, and 120 min after injection. Blood glucose during the tests was determined by Accu-ChekAdvantage II (Roche). The areas under the curves of blood glucose during ipITT and ipGTT were calculated from values of each mouse using the trapezoidal method [19].

### Intraperitoneal leptin tolerance test

The test was performed in 2 days, with an interval of 48 h between them. On the first day, after a 12-h overnight fasting, mice received an i.p. saline injection (10 ml/kg). Diet was weighed and offered to the mice immediately after injection. Food intake was measured every 2 h for 8 h. On the second day, after another overnight fast of 12 h, mice received an i.p. injection of leptin (120 mg/kg, 10 ml/kg). Food intake was measured as in the first day. The tolerance to leptin was evaluated by the difference in food intake between the two occasions [20].

### Hormones

Blood was collected in Eppendorf tubes containing heparin (1:1000). The plasma was obtained by centrifugation (2000 rpm for 10 min at 4°C) and stored at −80°C for determination of insulin (EZRMI-13K | Rat/Mouse Insulin ELISA - Merck Millipore), leptin (EZML-82K | Rat/Mouse Leptin ELISA - Merck Millipore), and adiponectin (EZMADP-60K | Mouse Adiponectin ELISA – Merck Millipore) [21].

### Western blotting

Both hypothalamus and liver were placed in anti-protease cocktail (Triton X-100 10%, 100 mM Tris (pH 7.5) containing 10 mM sodium pyrophosphate, 100 mM sodium fluoride, 10 mM EDTA, 10 mM sodium orthovanadate, 2 mM PMSF, and 1 µg/ml aprotinin) and homogenized with the aid of a Polytron (Kinematica, Switzerland). Then, the homogenate was centrifuged at 12000 rpm for 10 min. The supernatant was collected, and an aliquot was used for protein quantitation by the Bradford method, using a standard curve of a known concentration of albumin as reference. Samples containing 50 μg of protein were incubated at 100°C for 5 min in 20% of the volume of 5× Laemmli buffer (0.1% Bromophenol Blue, 0.5 M sodium phosphate, 50% glycerol, 10% SDS). For electrophoresis, a biphasic stacking gel (0.5 M Tris/HCl pH 6.8, 10% SDS, 30% Acrilamida/Bis, 10% APS) and resolution gel (1.5 M Tris/HCl pH 8.8, 10% SDS, 30% Acrilamida/Bis, 10% APS) was used. The run was performed at 120 V for approximately 180 min with running buffer (200 mM Trisma base, 1.52 mM glycine, 7.18 mM EDTA, and 0.4% SDS), diluted to 1:4. Samples were transferred on to a nitrocellulose membrane (Bio–Rad). The transfer was performed for 120 min at 120 V on ice, washed with transfer buffer (Trisma base 25 mM, glycine 192 mM, 20% methanol, and 0.02% SDS). After transfer, membranes were blocked with 5% albumin solution in TBS for 2 h at 4°C. Proteins related to the study were detected on the nitrocellulose membrane by overnight incubation at room temperature with the specific antibodies: Akt1/2/3 (Santa Cruz Biotechnology, sc81434), Thr^308^-p-Akt (Santa Cruz Biotechnology, sc16646), Y705 p-STAT 3 (Cell Signaling, #9131), STAT 3 (Cell Signaling #9139), and GAPDH (Santa Cruz Biotechnology, sc166545). Then the membrane was incubated with polyclonal anti-IgG (diluted 1:10000 in TBS with 2% albumin) followed by exposure for 2 h at room temperature. The immunoreactive bands were revealed by chemiluminescence after addition of the reagent Luminol (kit SuperSignaltm West Pico Chemiluminescent Substrate 34080) and detected with the Alliance 4.7 system (Uvitec, Cambridge, U.K.). Densitometry was performed using ImageJ software. For insulin signaling (Akt and p-Akt), mice were fasted for 12 h and then received insulin (100 μl, 10 IU i.p.). After 10 min, mice were killed and hypothalamus and liver were harvested [22].

### Real-time PCR array

Hypothalamus from C, H, and HE mice was removed, immediately snap-frozen, and stored at −80°C until RNA extraction. Then, the tissue was homogenized in Qiazol reagent (Qiagen) for 30 s using an Omni TH tissue homogenizer (Omni Inc., U.S.A.). Next, samples were centrifuged at 1500 rpm, and the total RNA content was isolated (RNeasy Microarray Tissue Mini Kit, catalog number 73304, Qiagen) according to the manufacturer’s instructions and quantitated by spectrophotometry (NanoDrop 2000, Thermo Scientific). cDNA synthesis was performed with 1 µg of total RNA through RT2 First Strand Kit (catalog number 330401, Qiagen) and gene expression was assessed by RT-PCR with the gene array system. This PCR array includes 84 obesity-related genes that are directly involved in the regulation of energy intake and expenditure (Mouse Obesity PCR Array, catalog number PAMM-017Z, Qiagen) [23]. Reading of the plates was carried out in the Step One Plus Real Time PCR System – Applied Biosystems and the data analyzed in the PCR Array System Data Analysis Software (Excel and Web based – SABioscience).

### Statistical analyses

Results are shown as mean ± S.E.M. Two-way or one-way ANOVA was employed followed by the Newman–Keuls post hoc test if necessary, using Statistica 12 software (StatSoft Inc.). Unpaired *t* test was used to compare the total number of wheel revolutions between the groups CE and HE. Significance was set at *P*<0.05.

## Results

The final body weight was significantly higher in both groups fed high-fat diet (H and HE) with a mean increase of 24% in H and HE groups compared with C and CE, with no effect of exercise. Accordingly, body weight gain was higher (139%) in H and HE groups, with no effect of exercise or interaction between the two factors. The total number of wheel revolutions during the entire experiment was similar between CE and HE groups. Mice of both high-fat diet fed groups consumed approximately 50% more energy at week 5 than mice of C and CE groups. At week 10, energy intake was similar (Table 1).

**Table 1:** General experimental parameters

	C	CE	H	HE
Initial body weight (g)	24.77 ± 0.63	24.22 ± 0.62	24.32 ± 0.34	25.06 ± 0.66
Final body weight (g)	29.54 ± 0.73	29.19 ± 0.73	37.57 ± 1.11*	35.21 ± 1.25*
Body weight gain (g)	4.77 ± 0.48	4.97 ± 0.54	13.24 ± 1.02*	10.15 ± 1.19*
Total wheel revolutions	—	202665 ± 14144	—	204329 ± 18610
Energy intake (kcal/day)				
Week 5	8.35 ± 0.63	9.03 ± 0.44	13.43 ± 0.79*	12.72 ± 0.47*
Week 10	10.06 ± 0.98	10.52 ± 0.76	10.26 ± 0.93	12.92 ± 1.01
Retroperitoneal fat pad weight (mg/g)	7.2 + 1.05	5.31 + 0.74^†^	16.48 ± 1.3*	12.16 ± 1.14*,^†^
Perigonadal fat pad weight (mg/g)	24.81 ± 2.78	19.9 ± 2.88^†^	55.27 ± 3.88*	41.06 ± 4.65*,^†^
Fast blood glucose (mg/dl)	148.22 ± 8.57	128.75 ± 6.41^†^	190.66 ± 11.44*	131.25 ± 4.62*,^†^
Insulin (ng/ml)	1.31 ± 0.32	1.40 ± 0.16	3.78 ± 0.70*	4.83 ± 0.83*
Leptin (ng/ml)	6.07 ± 1.34	3.09 ± 0.52^†^	23.04 ± 3.90*	11.20 ± 2.66*,^†^
Adiponectin (ng/ml)	30.88 ± 1.39	36.63 ± 2.76^†^	29.3 ± 1.5	34.27 ± 2.64^†^

Data are shown as mean ± S.E.M. Two-way ANOVA; *effect of diet, ^†^effect of exercise. Unpaired *t* test was used to compare the total number of wheel revolutions between the groups CE and HE; *P*<0.05. Abbreviations: CE, control diet fed group with free access to the wheel; HE, high-fat diet fed group with free access to the wheel. *n*=12–15, except for the hormones (*n*=8–10).

As for the results of final body weight, visceral fat depots weight was higher in H and HE in relation to C and CE groups. The increase was approximately 115% for perigonadal fat pad and 130% for retroperitoneal fat pad in high-fat diet fed groups compared with control diet fed groups. However, despite exercise had no effect in body weight, it reduced both fat pads in HE and CE groups. The perigonadal fat pad weight decreased by 26% in HE compared with H and 20% in CE compared with C. Retroperitoneal fat pad weight decreased by 27% in HE compared with H and 26.1% in CE compared with C (Table 1).

Fasting blood glucose was increased by high-fat diet. However, exercise was effective in decreasing fasting glycemia by 31% in HE compared with H and by 13% in CE compared with C. Circulating insulin was approximately 230% higher in high-fat diet fed groups (H and HE) than in control groups (C and CE). No effect of exercise was observed for insulin. Leptin was also increased in H and HE (approximately 277%) compared with C and CE groups, but wheel running decreased leptin by 52% in HE compared with H and 50% in CE compared with C. Plasma adiponectin levels were higher in the exercised (CE and HE) than in the non-exercised (C and H) groups. Voluntary running increased adiponectin by 17% in HE and 22% in CE compared with H and C, respectively (Table 1).

**Figure 1:**
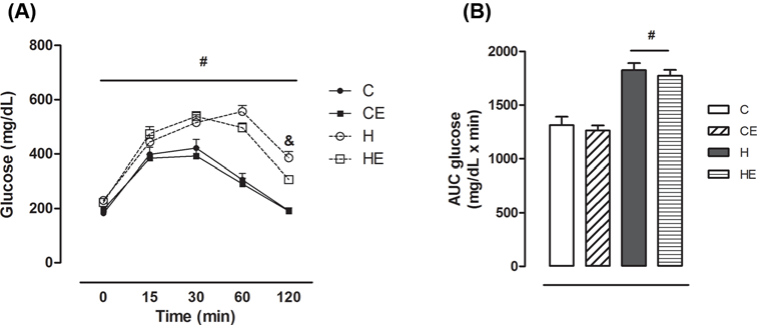
Glucose tolerance test carried out at the end of the experiement. (**A**) Blood glucose and (**B**) AUC blood glucose during ipGTT. Data are shown as mean + S.E.M. Two-way ANOVA; ^#^effect of diet, ^&^effect of exercise; *P*<0.05. C, control diet fed group (*n*=9); CE control diet fed group with free access to the wheel (*n*=8); H, high-fat diet fed group (*n*=10); HE, high-fat diet fed group with free access to the wheel (*n*=8).

Obesity is strikingly related to glucose intolerance and insulin resistance. Thus, the metabolic outcomes of high-fat diet and exercise were measured at the end of the experiment. As expected, the groups treated with high-fat diet developed glucose intolerance. As shown in Figure 1A, blood glucose in the groups H and HE was higher than in the C and CE groups during the entire test. HE group had lower glycemia 120 min after the glucose load administration compared with H group. The area under the curve (AUC) of blood glucose was approximately 28% higher in H and HE than in C and CE groups, with no effects of exercise (Figure 1B).

**Figure 2:**
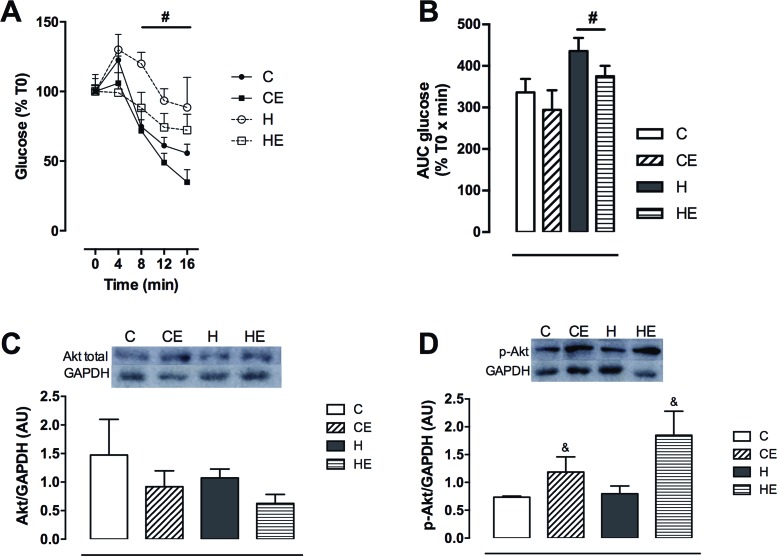
Insulin tolerance test and liver insulin signaling carried out at the end of the experiment. (**A**) Blood glucose and (**B**) AUC of blood glucose during ipITT, and (**C**) liver total Akt and (**D**) p-Akt expression. Data are shown as mean + S.E.M. Two-way ANOVA; ^#^effect of diet, ^&^effect of exercise; *P*<0.05. C, control diet fed group; CE, control diet fed group with free access to the wheel; H, high-fat diet fed group; HE, high-fat diet fed group with free access to the wheel. *n*=8-10 for ipITT and *n*=4–5 for liver protein expression.

High-fat diet also decreased insulin sensitivity, and no effect of exercise was detected. Blood glucose decay during ipITT was decreased as an effect of the high-fat diet (Figure 2A). AUC of normalized blood glucose was 29% higher in H and HE than in C and CE (Figure 2B). Peripheral insulin action was analyzed in liver. Total Akt expression was not affected by exercise or high-fat diet (Figure 2C). However, wheel running increased liver p-Akt. The exercised groups (CE and HE) had higher p-Akt content (38% in CE than in C and 57% in HE than in H) when compared with non-exercised groups (C and H) (Figure 2D).

**Figure 3:**
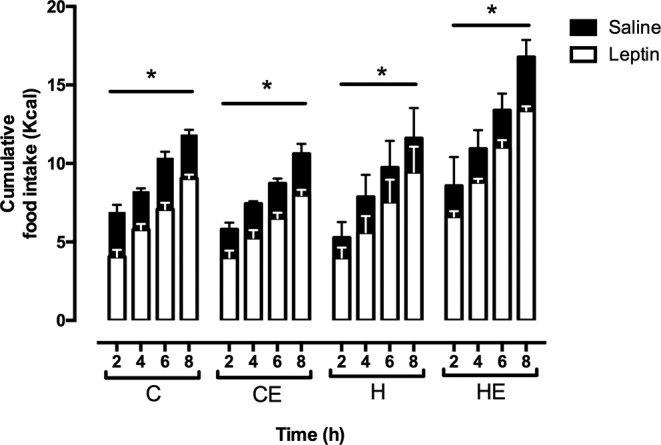
Cumulative food intake of mice after saline and leptin administration Data are shown as mean + S.E.M. The effect of leptin (*) was analyzed by repeated-measure one-way ANOVA; *P*<0.05. C, control diet-fed group; CE, control diet fed group with free access to the wheel; H, high-fat diet fed group; HE, high-fat diet fed group with free access to the wheel. *n*=4–5.

Leptin is a satiety signal acting at hypothalamus. To evaluate its central action, a leptin tolerance test was performed. Cumulative food intake increased as an effect of time in all groups. Compared with saline injection, intraperitoneal leptin administration inhibited food intake in all groups (Figure 3).

**Figure 4:**
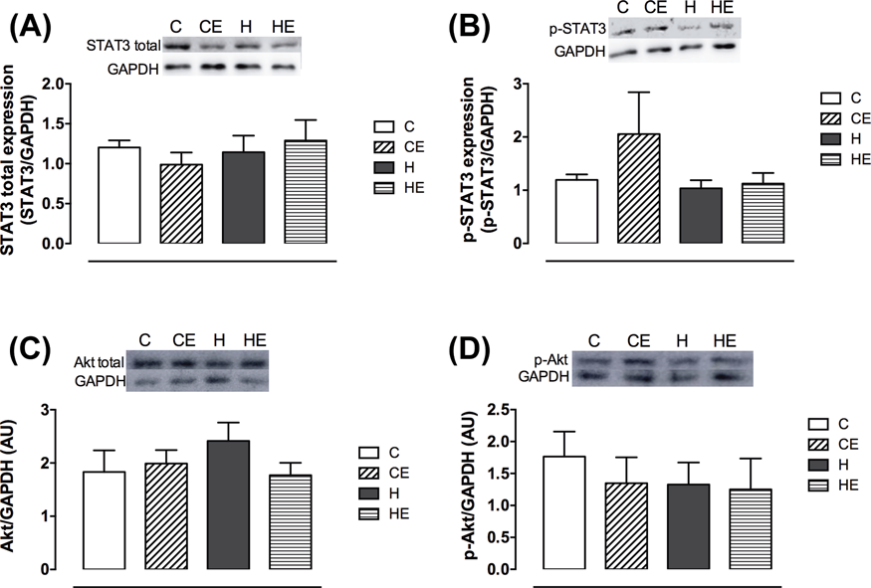
Hypothalamic leptin and insulin signaling Hypothalamic expression of (**A**) total and (**B**) p-STAT3, (**C**) total and (**D**) p-Akt. Data are shown as mean ± S.E.M. Two-way ANOVA; *P*<0.05. C, control diet fed group (*n*=5); CE, control diet fed group with free access to the wheel (*n*=4); H, high-fat diet fed group (*n*=5); HE, high-fat diet fed group with free access to the wheel (*n*=4).

Hypothalamic insulin and leptin signaling were also evaluated. Total STAT3 and p-STAT3 as well as total Akt and p-Akt (Figure 4A–D) expression were similar amongst the groups.

Using real-time PCR array, we evaluated the expression of genes related to energy homeostasis in hypothalamus. High-fat diet modulated the expressions of 14% of the 84 analyzed targets. Of these, only one was not differentially expressed in HE compared with H (Table 2).

**Table 2:** Expression of genes related to energy homeostasis in hypothalamus

Gene symbol	Fold change			Gene name
	H compared with C	HE compared with C	HE compared with H	
*Adrb1*	0.53	0.58	1.08	Adrenergic receptor β 1
*Bdnf*	0.53	0.57	1.08	Brain-derived neurotrophic factor
*Calcr*	0.63	0.57	ns	Calcitonin receptor
*Cntfr*	0.53	0.57	1.08	Ciliary neurotrophic factor receptor
*Crhr1*	1.06	1.14	1.08	Corticotropin releasing hormone receptor 1
*Htr2c*	1.07	1.15	1.08	5-hydroxytryptamine receptor 2C
*Il-1α*	1.06	0.57	0.47	Interleukin-1α
*Il-1r1*	0.54	0.58	1.08	Interleukin 1 receptor type 1
*NPY1r*	1.06	1.15	1.08	Neuropeptide Y receptor Y1
*Ppar-γ*	0.53	1.15	2.17	Peroxisome proliferator activated receptor γ
*Ptpn1*	1.07	1.15	1.08	Protein tyrosine phosphatase non-receptor type 1
*Zfp91*	1.07	1.16	1.08	Zinc finger protein 91

All genes displayed above are differentially expressed (*P*<0.05). One-way ANOVA and Newman–Keuls post hoc test. C, control diet-fed group; H, high fat diet-fed groups; HE, high fat diet-fed group with free access to the wheel; *n*=4. Abbreviation: ns, not significant.

## Discussion

As found previously by us and others [4,6,10,11], free access to running wheel did not lead to significant body weight loss. To explain the reasons for the lack of effect of voluntary running to induce negative energy balance, Brown et al. (2012) [17] proposed the existence of an undefined distance threshold which must be overcome to protect from DIO. Moreover, compensatory mechanisms induced by voluntary running, including decrease in spontaneous physical activity and increase in energy intake [6], are also probably involved. However, beneficial molecular and metabolic changes might still be happening despite no changes in body weight. A decrease in visceral and total abdominal fat may occur in the absence of weight reduction [24]. Likewise, CRF is inversely related to inflammatory markers even after correcting for body mass index [25], and high CRF is associated with a substantial reduction in metabolic syndrome markers independent of visceral and subcutaneous fat [26].

Peripherally, glucose intolerance is the main outcome arising from insulin resistance [27]. Mice fed a high-fat diet are hyperinsulinemic and severely glucose intolerant and insulin resistant [28–30]. Chronic positive energy balance results in expansion of adipose tissue and consequent macrophage infiltration, leading to the production of proinflammatory mediators and activation of the JNK and IKKβ/NFκB signaling pathways. Once activated, both JNK and IKK down-regulate insulin signaling [31,32]. Exercise is known to improve insulin resistance [33–35]. Even though glucose uptake during ITT was not affected by wheel running, liver Akt phosphorylation was increased in exercised mice despite no change in body weight. As insulin suppresses liver glucose production [36,37], this is consistent with the reduced fast blood glucose in exercised (CE and HE) compared with non-exercised groups (C and H).

Similar to insulin, the activation of JNK and IKKβ/NFκB by proinflammatory cytokines also leads to reduced leptin signaling [38]. Accordingly, hypothalamic insulin and leptin resistance is a hallmark of obesity and leads to hyperphagia and reduced locomotion [39–41]. Thus, we investigated whether exercise without weight loss can modulate insulin and leptin signaling in hypothalamus. We did not find alteration in the amount of p-Akt and STAT3, which is consistent with the similar energy intake amongst the groups at the end of the study, despite the excessive energy consumption at week 5. Moreover, intraperitoneal leptin administration suppressed food intake in all mice, including those in both high-fat diet fed groups. However, one needs to be cautious to interpret these data because even though mice received the same dose of intraperitoneal leptin, H and HE mice had already a plasma leptin concentration approximately four-times higher than C and CE, respectively. With respect to insulin, the plasma level of this hormone was approximately three-times higher in H and HE compared with C and CE.

High-fat diet also changed the expression of several hypothalamic genes involved in energy homeostasis in both H and HE groups. Some of the modulated genes interfere negatively with leptin and insulin signaling, favoring a positive energy balance. We found a down-regulation of interleukin 1 receptor type 1 (Il-1r1) and an up-regulation of protein tyrosine phosphatase non-receptor type 1 (Ptpn1). Whereas knockout of *Il-1r1* gene causes leptin resistance and obesity [42,43], Ptpn1 knockout results in negative energy balance, protection from weight gain and improved insulin sensitivity [44–46]. Additionally, the expression of the proinflammatory cytokine interleukin-1α (Il-1α) was also up-regulated in high-fat diet fed mice. As described previously, a proinflammatory state is known to occur in DIO mice and to be associated with hypothalamic insulin and leptin resistance [31,32]. Accordingly, Il-1α is higher in obese than in slim mice [47,48]. As discussed above, we did not find alteration in hypothalamic insulin and leptin signaling in H and HE mice. However, the same level of Akt and STAT3 phosphorylation was obtained in high-fat diet fed groups with a much higher plasma concentration of insulin and leptin. This suggests some degree of impairment on the action of both hormones, which is in-line with the changes in gene expression caused by high-fat diet that we observed.

Other genes modulated by the high-fat diet might have equally contributed to the obese phenotype. Calcitonin receptor (Calcr), ciliary neurotrophic factor receptor (Cntfr) and brain-derived neurotrophic factor (Bdnf) were all down-regulated. Activation of the former has been shown to decrease food intake and weight gain [52], while the receptor for the Cntf anoretic cytokine induces long-lasting weight reduction that seems to be related to promotion of hypothalamic neurogenesis [53]. Bdnf seems to reduce body weight by decreasing food intake and increasing EE and locomotion [54–58]. Importantly, Bdnf has a crucial role not only in energy balance but also in development, maintenance, and plasticity of the nervous system [59,60], and its down-regulation on hypothalamus highlights the harmful effect of high-fat diet. With respect to Neuropeptide Y receptor Y1 (NPY1r), which is involved in the process of feeding stimulated by NPY [49], its modulation was very subtle. Knocking out NPY1r reduces food intake, body weight, and adipose tissue in mice [50,51].

At the same time, the modulation of the genes mentioned above supports a positive energy balance, there seems to be an unsuccessful attempt of restoring homeostasis. Corticotropin releasing hormone receptor 1 (Crhr) and 5-hydroxytryptamine receptor 2C (Htr2C) gene expression was up-regulated by high-fat diet. Crhr is reported to reduce food intake under stressful circumstances [61,62] and loss of Htr2C resulted in hyperphagia in mice [63,64]. Furthermore, high-fat diet down-regulated peroxisome proliferator activated receptor γ (Ppar-γ) and adrenergic receptor β 1 (Adrb1) gene expression. Hypothalamic overexpression of Ppar-γ causes positive energy balance by limiting thermogenesis and increasing food intake [65,66]. With respect to Adrb1, in ob/ob mice the development of metabolic syndrome seems to be related to increased hypothalamic norepinephrine activity. If this is also true for DIO, down-regulation of Adrb1 might act as a compensatory mechanism to the increased noradrenergic activity. At last, the role of hypothalamic zinc finger protein 91 (Zfp91) is less clear but it could be implicated in energy homeostasis [67].

Of note, exercise without weight reduction only marginally modulated gene expression, except for Il-1α and Ppar-γ, consistent with the lack of effect of exercise on energy intake. As a proinflammatory cytokine, the down-regulation of *Il-1α* gene in HE related to H can be seen as a positive result. Ppar-γ, on the other hand, was down-regulated in H but up-regulated in HE mice, and given its role in energy homeostasis [65,66], this gene could be at least partially involved in the compensatory behaviors that prevent the exercise-induced negative energy balance. In old ApoE^−/−^ mice fed a high-cholesterol diet exercise had marked effects on brain, including decreased inflammation and oxidative stress, as shown by MRI. However, these effects were seen only if dietary intake was controlled whereas *ad libitum* feeding, as used in our study, abrogated the benefits of exercise [68,69], which could be related to such a discrete effect of exercise on hypothalamic gene expression we observed.

Importantly, despite no change in body weight, voluntary exercise decreased fat depots. Plasma leptin correlates with adiposity [70–72]. Hence, leptin concentration was higher in both high-fat diet fed groups but as for the adipose depots, exercise reduced leptin concentration. Besides, as found in other studies [35,73], exercise increased adiponectin concentration. In addition to its anti-inflammatory action, adiponectin has antidiabetic, antiatherogenic, and insulin-sensitizing properties [74]. The latter might help to explain the improved liver insulin signaling and fast blood glucose in the exercised groups (CE and HE). Thus, even though it is known that weight loss is desirable and is associated with improvements in many cardiometabolic risk factors in overweight and obese individuals [75], our results indicate that physical activity must be encouraged even in the absence of body weight reduction. In-line with this, the benefits of exercise on mortality risk irrespective of adiposity has already been demonstrated even in high-risk individuals with prediabetes [76].

In conclusion, exercise caused important peripheral positive changes (decreased fast glycemia and fat depots; increased liver insulin signaling and adiponectin concentration) even without weight loss. Centrally, however, the effects of exercise were more discrete. Thus, despite reducing body weight can bring additional benefits, the effects of exercise must not be overlooked when body weight reduction is not achieved.

## Abbreviations

Adrb1: adrenergic receptor β 1
AUC: area under the curve
Bdnf: brain-derived neurotrophic factor
CE: control diet fed group with free access to the wheel
Crhr: corticotropin releasing hormone receptor 1
CRF: cardiorespiratory fitness
DIO: diet-induced obesity
EE: energy expenditure
HE: high-fat diet fed group with free access to the wheel
Htr2C: 5-hydroxytryptamine receptor 2C
IKKβ: inhibitor of nuclear factor kappa B kinase subunit beta
Il-1r1: interleukin 1 receptor type 1
Il-1α: interleukin-1α
ipGTT: intraperitoneal glucose tolerance test
ipITT: intraperitoneal insulin tolerance test
JNK: c-Jun N-terminal kinase
NFκB: nuclear factor kappa B
NPY1r: neuropeptide Y receptor Y1
Ppar-γ: peroxisome proliferator activated receptor γ
Ptpn1: protein tyrosine phosphatase non-receptor type 1
